# Supplementary material for: Detection of Polonium-210 in Environmental, Biological and Food Samples: A Review
Source: Molecules. 2023 Aug 27;28(17):6268. doi: 10.3390/molecules28176268 (PMC10488615; doi:10.3390/molecules28176268)
Supplement: Supplementary file 1 [file molecules-28-06268-s001.zip › molecules-2576473-SI.pdf]

Supporting Information

Detection of Polonium-210 in Environmental, Biological and Food Samples: A Review

Lei Zhou [1,†], Rui Wang [1,2,†], Hong Ren [1], Peng Wang [1] and Yiyao Cao [1,*]

[1] Department of Occupational Health and Radiation Protection, Zhejiang Provincial Center for Disease Control and Prevention, Hangzhou 310051, China; zhoulei@cdc.zj.cn (L.Z.); w9906070012@163.com (R.W.); hren@cdc.zj.cn (H.R.); pwang@cdc.zj.cn (P.W.)

[2] School of Public Health, Suzhou Medical College, Soochow University, Suzhou 215123, China

* Correspondence: yycao@cdc.zj.cn; Tel.: +86-(0571)-87115089

† These authors contributed equally to this work.

Table of content

Figure S1…..…………………………………...……..........……………………...…........................S2

Figure S2…..…………………………………...……..........……………………...…........................S2

Figure S3…..…………………………………...……..........……………………...…........................S3

Chemical Purification	Reagents	Advantage	Disadvantage
**Solvent extraction**	● Isopropyl ether;<br>● Methyl isobutyl ketone;<br>● Diisopropyl ketone;<br>● Tributyl phosphate;<br>● DDTC;<br>● TOPO.	● Easy operation;<br>● Large selection of reagents;<br>● Suitable for a wide range of hydrochloric acid concentrations.	● Generates mixed radioactive waste;<br>● Third phase formation.
**Ion exchange chromatography**	● Dowex-1;<br>● Dowex-2;<br>● Bio-rad AG1-X4;<br>● Bio-Rad AG50Wx8.	● Low interference by other nuclides;<br>● Large selection of reagents;<br>● High selectivity for polonium.	● Complexity of operational steps;<br>● Labour-intensive.
**Extraction chromatography**	● Sr resin;<br>● DGA resin;<br>● HRA resin.	● High selectivity for polonium;<br>● Faster exchange kinetics than anion exchange;<br>● Simple operation steps;<br>● Reducing the volume of highly radioactive waste.	● Complexity of operational steps;<br>● Labour-intensive;<br>● Longer experimentation time.

Figure S1. The advantages and disadvantages of the different chemical purification for ^{210}Po.

Source preparation		Advantage	Disadvantage
**Spontaneous deposition**		● Better selectivity for polonium;<br>● Higher choice of deposition materials;<br>● Does not require power for use.	● Need to remove interfering ions;<br>● Longer deposition times.
**Electrodeposition**		● Large selection of materials;<br>● Better selectivity for polonium;<br>● Shorter depositiont time.	● Requires power for use;<br>● Complicated procedure.
**Micro-precipitation**	CuS micro-precipitation	● Faster source preparation;<br>● No heating required;<br>● Suitable for bulk sample processing.	● Poor recovery stability;<br>● Low acid resistance.
	Te micro-precipitation	● No heating required;<br>● Suitable for large number of samples;<br>● Higher acid resistance;<br>● Good selectivity for polonium.	● Incomplete reduction of Te(IV) to Te under acidic conditions;<br>● Not widely used.
	$BiPO_4$ micro-precipitation	● Reduced source production time;<br>● No heating required;<br>● Suitable for large volume urine samples.	● Interference by other alpha nuclides;<br>● Requires purification steps.

Figure S2. The advantages and disadvantages of the different source preparation for ^{210}Po.

Measurements		Advantage	Disadvantage
Alpha spectrometry	Alpha spectroscopy with tracer	• Simple operation;<br>• Low detection limits;<br>• High energy resolution;<br>• Effective separation from other alpha nuclides.	• Complex pretreatment steps are required;<br>• Tracers are difficult to obtain;<br>• Tracers contamination.
	Total alpha counting	• No tracer is used;<br>• Matrix elements are not separated;<br>• Suitable for near-field measurements.	• Accurate recovery of samples cannot be determined.
Liquid scintillation counting (LSC)	Unoptimized LSC	• No contamination of the detector chamber;<br>• High detection efficiency.	• Poor energy resolution;<br>• High detection limits.
	Photon electron rejecting liquid alpha spectroscopy	• Low detection limits;<br>• Smaller sample volume;<br>• Simple operation without complex pretreatment.	• High-purity phosphoric acid is more difficult to obtain;<br>• Separation of other nuclides.
Large area screen grid spectrometry		• Low detection limits;<br>• Simple sample preparation process;<br>• Simultaneous determination of multiple nuclides.	• High requirements for sample particles;<br>• Large area source stability is not good;<br>• Not widely used.

Figure S3. The advantages and disadvantages of the different radioactivity measurements for ^{210}Po.
